# The Role of the Wnt/β-Catenin Signaling Pathway in the Pathogenesis and Treatment of Spinal Cord Injury: A Review of the Latest Experimental Data

**DOI:** 10.7759/cureus.87836

**Published:** 2025-07-13

**Authors:** Grigoria Fellouri, Konstantinos Savvas, Niki Tsoutsi, Efstathios Kourtis, Ilias Fanourgiakis, Panagiotis Lepetsos, Elias Vasiliadis

**Affiliations:** 1 Orthopedics, KAT General Hospital, Athens, GRC; 2 Orthopedics, Henry Dunant Hospital Center, Athens, GRC; 3 Orthopedics, Athens Medical Center, Athens, GRC; 4 Orthopedic Surgery, KAT General Hospital, School of Medicine, National and Kapodistrian University of Athens, Athens, GRC

**Keywords:** management, pathogenesis, spinal cord injury, wnt, β-catenin

## Abstract

Spinal cord injury (SCI) is a debilitating condition with a high incidence, leading to irreversible motor, sensory, and autonomic dysfunctions. Despite significant advances in understanding the pathophysiology of SCI, effective therapeutic strategies for regeneration remain elusive. The Wnt/β-catenin signaling pathway has emerged as a key player in various developmental and pathological processes, including tissue regeneration, neuroprotection, and inflammation, all of which are crucial in SCI. The aim of this paper is to review the current understanding of the Wnt/β-catenin signaling pathway in the pathogenesis of SCI and its potential as a therapeutic target for improving recovery after SCI. The Wnt/β-catenin signaling pathway has a distinctive role in SCI pathogenesis, encompassing regulation of inflammation, apoptosis, glial scar, axonal repair and regeneration, and angiogenesis. Many treatment modalities, such as pharmaceutical administration, stem cell transplantation, gene therapy, electric stimulation, and the use of biomaterials and exosomes, exert beneficial effects in SCI animal models by interacting with the Wnt/β-catenin signaling pathway. The Wnt/β-catenin signaling pathway plays a multifaceted role in the pathogenesis of SCI and offers a promising avenue for therapeutic intervention. Its effects depend on the specific Wnt ligands involved, the injury stage, and the cellular environment. Precisely modulating Wnt signaling could be a promising therapeutic strategy for enhancing spinal cord repair and functional recovery after injury. Although significant progress has been made, further studies are needed to improve our understanding of its context-dependent effects and to address therapeutic challenges.

## Introduction and background

Spinal cord injury (SCI) is a catastrophic event that results in permanent disability and affects millions of people worldwide. SCI is often caused by trauma, leading to varying degrees of damage to the spinal cord, disrupting its structure and function. Despite advances in acute management, such as corticosteroids and surgical interventions, effective therapies for repairing the damaged spinal cord and promoting functional recovery remain largely ineffective [1].

Primary damage in SCI occurs after direct mechanical trauma, including contusion, compression, or laceration of the spinal cord, leading to disruption of neural and vascular structures. Axonal shearing occurs when neurons are physically stretched or severed, resulting in the loss of connectivity. Hemorrhage and ischemia follow, reducing blood flow and oxygen supply to the affected region, which can trigger widespread cell death. Since primary damage is irreversible, it initiates a cascade of secondary injury mechanisms that further exacerbate the damage [2,3].

Secondary damage develops over a period of hours to weeks following the initial injury and significantly worsens neuronal tissue loss and functional impairment. Inflammation plays a crucial role, as activated microglia and infiltrating immune cells release pro-inflammatory cytokines such as TNF-α and IL-1β, leading to further cell damage [4]. Oxidative stress arises due to an excess of free radicals, which cause lipid peroxidation, protein degradation, and DNA damage. Excitotoxicity occurs when excessive glutamate release overstimulates neurons, resulting in calcium overload and subsequent neuronal death [1]. Apoptosis further contributes to tissue degeneration, as neurons and oligodendrocytes undergo programmed cell death, leading to extensive white matter loss. Another critical process in secondary damage is glial scar formation, where reactive astrocytes proliferate and secrete extracellular matrix proteins [5]. While the glial scar helps contain the injury site and prevent further damage, it also serves as a physical and chemical barrier to axonal regeneration, ultimately limiting recovery. Because secondary damage significantly amplifies the effects of primary injury, it represents a key target for therapeutic interventions aimed at reducing neuroinflammation, oxidative stress, and inhibitory scarring [3,4].

The Wnt signaling pathway is a complex network of proteins that play a central role in regulating cell fate, adhesion, polarity, and migration. There are two major branches of the Wnt signaling pathway: the canonical (β-catenin-dependent) pathway and the non-canonical (β-catenin-independent) pathway [6]. In the canonical pathway, the binding of Wnt ligands (such as Wnt1, Wnt3a, and Wnt7) to the frizzled receptor family triggers a cascade of intracellular events. In the absence of Wnt signaling, β-catenin is phosphorylated and degraded by a destruction complex consisting of APC, axin, and GSK-3β. Upon Wnt binding, the destruction complex is inhibited, allowing β-catenin to accumulate in the cytoplasm and translocate to the nucleus. In the nucleus, β-catenin interacts with transcription factors, including T-cell factor and lymphoid enhancer-binding factor, to activate the expression of target genes involved in cell proliferation, survival, differentiation, and tissue regeneration [7]. The non-canonical Wnt/β-catenin signaling pathway operates independently of β-catenin and is primarily involved in regulating cell movement, polarity, and intracellular calcium signaling. It consists of two main branches: the Wnt/Planar Cell Polarity pathway and the Wnt/Ca²⁺ signaling pathway. Unlike the canonical Wnt/β-catenin pathway, which primarily regulates gene transcription, the non-canonical Wnt signaling pathways focus on cytoskeletal dynamics, cell polarity, and intracellular signaling, making them essential for tissue organization, development, and disease progression [8].

In recent years, the Wnt/β-catenin signaling pathway has garnered attention for its role in various cellular processes, including cell proliferation, differentiation, and survival, which are crucial in injury and repair mechanisms. Initially studied in the context of development and cancer, the Wnt/β-catenin pathway has been implicated in neurogenesis, tissue regeneration, and neuroprotection [9]. Recent evidence suggests that it also plays a critical role in the pathophysiology of SCI and spinal cord repair. Dysregulation of the Wnt/β-catenin pathway can lead to impaired cellular responses to SCI, exacerbating damage and hindering recovery [6]. This paper aims to explore the role of the Wnt/β-catenin pathway in the pathophysiology of SCI and its potential in SCI management through therapeutic interventions.

## Review

This is a simple literature review using the PubMed internet database. Papers were searched with the use of the following keywords: (“Wnt” OR “β-catenin”) AND (“spinal cord injury”). Experimental or in vitro studies addressing the role of the Wnt/β-catenin signaling pathway in the pathogenesis and treatment of spinal cord injuries were sought. Clinical studies, systematic reviews, case reports, and studies in languages other than English were excluded.

Detection of the Wnt/β-catenin signaling pathway after SCI

mRNA encoding most Wnt ligands and soluble inhibitors is constitutively expressed in the healthy adult spinal cord [10,11]. The majority of the known Frizzled receptors were expressed in specific spatial patterns in the normal spinal cord [12].

After SCI, Wnt signaling is activated via the Wnt/β-catenin pathway. SCI induces a time-dependent increase in Wnt mRNA expression from six hours until one month post-injury and a narrow peak in the expression of soluble Wnt inhibitors between one and three days post-injury. Following SCI, Wnt1 and Wnt5a were induced broadly in the spinal cord gray matter and in reactive astrocytes, while Wnt4 was induced in areas closer to the lesion than Wnt1 and Wnt5a [13,14]. The frizzled mRNAs and proteins were expressed after SCI, and frizzled 5 was expressed in reactive microglia/macrophages three to 14 days following SCI [12].

After an initial decrease by 1 day, LRP6, a Wnt co-receptor, and β-catenin sustain a dramatic change, from a homogeneous expression pattern in the grey matter to a disorganized injury-induced pattern [15]. DKK1 and Wif1 are potent inhibitors of the Wnt/β-catenin signaling pathway. A significant upregulation of Dkk1 was detected at 24 hours following SCI, peaking at three days after SCI and remaining elevated until 42 days after SCI [11]. Wif1 is increased after SCI in animal models [10].

Sclerostin is an osteocyte-derived glycoprotein, an inhibitor of bone formation that negatively regulates Wnt signaling after connecting to the LRP5/LRP6 co-receptors. After SCI, in response to mechanical unloading, its levels are initially increased [16]. The Wnt pathways were down-regulated in osteoblasts from spinal cord-injured rats as a result of reduced bone formation [17]. SCI was found to attenuate Wnt signaling in sublesional tibiae [18]. LGR5, a key regulator of the Wnt/β-catenin signaling pathway, was co-expressed with GFAP after SCI in rats [19].

The role of the Wnt/β-catenin signaling pathway in SCI pathogenesis

The Wnt/β-catenin pathway plays a multifaceted role in both promoting and inhibiting various aspects of SCI pathophysiology.

Regulation of Inflammation and Immune Response

The Wnt/β-catenin pathway is involved in regulating the immune response in SCI. Studies have shown that β-catenin can modulate the activation of microglia and astrocytes, influencing the production of inflammatory mediators. In particular, β-catenin signaling can enhance the release of pro-inflammatory cytokines such as TNF-α, IL-1β, and IL-6, which can promote neuroinflammation [20,21].

Activation of the Wnt/β-catenin signaling pathway attenuates microglia-induced neuronal damage by shifting microglia to an anti-inflammatory phenotype. The activation of the canonical Wnt/β-catenin pathway mitigates microglial proliferation and suppresses inflammation, while the activation of the non-canonical Wnt/β-catenin pathway elicits microglial activation and promotes an inflammatory response [20,21]. Polarization and migration of reactive astrocytes in the subacute phase of SCI are promoted by the activation of the Wnt/β-catenin signaling pathway by M2 macrophages, which participate in the formation of glial scars, effectively inhibiting the inflammatory response and limiting the spread of the inflammation into the surrounding tissue [22,23]. Sirtuin 1 is an NAD⁺-dependent deacetylase enzyme that plays a crucial role in cellular metabolism, aging, stress resistance, and longevity. According to Lu et al., sirtuin1 has a neuroprotective role by inhibiting microglial activation through suppression of the Wnt/β-catenin signaling pathway after SCI [24].

Regulation of Apoptosis

The modulation of cellular apoptosis by the non-canonical Wnt/β-catenin signaling pathway is an important mechanism in adapting to the SCI microenvironment and regulating cellular homeostasis. Following SCI, intracellular calcium levels may increase, leading to the activation of various calcium-dependent effector molecules through the non-canonical Wnt/β-catenin signaling pathway. After SCI, Wnt-3a enhances functional outcome in the SCI region through autophagy activation through the inhibition of the mTOR signaling pathway [25,26]. Wnt-3a may significantly suppress neuronal apoptosis after SCI, decrease motor neuronal loss in the spinal cord, and augment the repair of damaged tissue [23,26,27].

MicroRNA-137 is important in delaying neuronal apoptosis through the KDM4A/SFRP4/Wnt3a/β-catenin axis [28,29]. After SCI, Circ-Ctnnb1 decreases neuronal cell apoptosis by modulating the microRNA-205-5p/Ctnnb1/Wnt2b axis [30]. Sirtuin 1 is negatively correlated with β-catenin in SCI, which stimulates the apoptosis of motor neuron cells [31].

Regulation of Glial Scar Formation and Tissue Repair

The Wnt/β-catenin pathway has been implicated in regulating the formation of the glial scar. In some cases, β-catenin signaling has been shown to promote the differentiation of astrocytes into a scar-forming phenotype, which could exacerbate tissue damage [32]. In adult zebrafish with SCI, Dkk1b overexpression mitigates locomotor recovery, axon regeneration, and glial bridge formation in the injured spinal cord through inhibition of Wnt/β-catenin signaling [33]. However, other studies suggest that Wnt signaling can promote the repair of the glial scar by enhancing the differentiation of oligodendrocyte precursor cells into mature oligodendrocytes, which are critical for remyelination. Wnt3a at the site of injury may promote the differentiation of oligodendrocyte precursor cells, inducing myelin sheath repair in axonal injuries [34]. However, blocking the Wnt/β-catenin pathway mitigates axonal regeneration and the formation of glial bridges in the damaged spinal cord, preventing motor functional improvement [33]. Throughout SCI, Wnt1 orchestrates the activation of ependymal cells from their quiescent state and their subsequent proliferation, a fact that contributes to SCI repair, promoting the stability and structural integrity of the spinal cord environment in the injured site [35].

Regulation of Axonal Regeneration and Neuroprotection

The Wnt/β-catenin pathway has been shown to have a critical role in promoting axonal growth and regeneration in the injured spinal cord [36].

In animal models of SCI, activation of the Wnt/β-catenin signaling pathway has been shown to promote axonal sprouting and increase the survival of injured neurons [36]. This regenerative effect is thought to be mediated by the upregulation of genes involved in cell survival, neuronal plasticity, and synaptic formation. In a mouse model of SCI, the miR-124-Neat1-Wnt/β-catenin signaling axis has been found to be involved in regulating the cell function of spinal cord progenitor cells [37]. Wnt5a overexpression reduces neuronal cell density, the accumulation of NG2+ glial precursors, and the descending serotonergic innervation in the affected areas, along with impairment of motor and bladder function recovery after SCI [38]. Long non-coding RNA H19 regulates osteogenic differentiation of BM-MSCs in post-SCI osteoporosis through the miR-29b-3p/DKK1 axis and by directly inhibiting the Wnt/β-catenin signaling pathway [39].

Furthermore, the Wnt/β-catenin pathway may promote the reprogramming of glial cells into a more neurogenic phenotype, which could facilitate the repair of damaged spinal cord tissue. In SCI, the canonical Wnt/β-catenin signaling pathway also plays an important role in the stimulation of neural stem cells’ proliferation and differentiation, along with axonal regeneration and neurite outgrowth. Wnt3a could significantly enhance the recovery of motor function by reducing the motor neuronal loss in the anterior horn of the spinal cord after SCI [23]. By reducing β-catenin levels, RAGE blockade attenuated the number of surviving neurons and decreased the area of spared white matter around the epicenter in SCI models [40]. Netrin-1 is a secreted protein that plays a crucial role in cell signaling, particularly in axon guidance, cell migration, and tissue development. Νetrin-1 has been found to exert its neuroprotective effect by activating the Wnt signaling pathway after SCI [41].

Ryk is an atypical Wnt receptor involved in non-canonical Wnt signaling. Without any enzymatic activity, Ryk plays a crucial role in Wnt-mediated processes, particularly in neuronal development, axon guidance, regeneration, and cancer progression. Wnt/Ryk signal mediates the inhibition of corticospinal axon growth in the adult spinal cord [14]. According to Liu et al., during SCI, local activation of the Wnt/Ryk signaling transduction system triggers the retraction of corticospinal tract axons from the lesion and attenuates the growth of proximal axon segments [13]. In embryonic mice, Ryk has been found to inhibit axonal growth in response to Wnt5a, and Wnt5a attenuates dendrite growth independently of Ryk [42]. Despite the fact that the non-canonical Wnt/β-catenin pathway seems to mitigate axonal regeneration after SCI, it participates in the post-SCI neural circuit reconstruction [43]. The equilibrium between the canonical Wnt/β-catenin pathway and the non-canonical pathway is crucial for axonal repair after SCI.

Regulation of Angiogenesis

Ensuring vascular structural integrity is essential for maintaining the equilibrium of the microenvironment of the blood-spinal fluid. Reactivating the Wnt/β-catenin pathway through external stimulation can prompt endothelial cells to release vascular growth factors, thereby supporting vascular regeneration and neuronal recovery after SCI [44]. Ozone treatment causes an increase in blood vessel density and stem cell proliferation and differentiation after SCI in rodents by increasing β-catenin levels [45]. Regarding the non-canonical Wnt/β-catenin pathway, SCI triggers macrophage activation and Wnt5a release [38]. Thus, the non-canonical Wnt/β-catenin pathway plays a crucial role in tissue angiogenesis and vascular remodeling. Activating both the canonical and non-canonical Wnt/β-catenin signaling pathways post-SCI can contribute to stabilizing the blood-spinal cord barrier and enhancing angiogenesis.

Regulation of Chronic/Neuropathic Pain

Research has shown that both Wnt signaling pathways are upregulated in SCI neuropathic pain and the dorsal horn of the spinal cord. This upregulation contributes to heightened neuronal inflammation and promotes the remodeling and strengthening of synaptic connections, ultimately intensifying pain signaling.

Studies in animal models have identified the activation of non-canonical Wnt/β-catenin signaling pathways in the spinal dorsal horn, which increase chronic inflammation levels, contributing to hyperalgesia [43]. During the later stages of SCI, Wnt10a/β-catenin regulates kindlin-1-mediated astrocyte activation. Knockdown of Wnt10a may decrease hyperalgesia and allodynia after SCI [46].

Role of MicroRNAs

MicroRNAs are small, non-coding RNA molecules that regulate gene expression at the post-transcriptional level. MicroRNA-25 protects PC-12 cells against oxidative stress through activation of Wnt/β-catenin signaling [47]. MicroRNA-124 improves SCI in rats by activating the Wnt/β-catenin signaling pathway [48]. MicroRNA-137 targeted KDM4A and then downregulated SFRP4 to ameliorate SCI in a Wnt/β-Catenin-dependent manner [27]. MicroRNA-219-5p inhibitor has a protective role in SCI via regulating the LRH-1/Wnt/β-catenin signaling pathway [49]. MicroRNA-200b-3p is important for Wnt5a-induced neural stem cell differentiation into neurons to promote motor functional and histological recovery after SCI [50]. MicroRNA-872-5p affected neural stem cells' proliferation by targeting FOXO3a to increase the expression of β-catenin [29].

The role of Wnt/β-catenin in SCI management

Given the crucial role of the Wnt/β-catenin pathway in SCI and repair, modulating this pathway has emerged as a potential therapeutic strategy for enhancing recovery after SCI. However, the dual nature of Wnt signaling - both promoting regeneration and potentially exacerbating inflammation and glial scarring - presents a significant challenge in designing targeted therapies.

One approach to promoting recovery after SCI is to activate the Wnt/β-catenin pathway to stimulate neurogenesis, axonal regeneration, and tissue repair. This could be achieved through the use of small molecules that mimic the effects of Wnt ligands or inhibit the destruction complex, thereby stabilizing β-catenin. Preclinical studies have demonstrated that pharmacological activation of the Wnt/β-catenin pathway can enhance axonal growth, promote oligodendrocyte differentiation, and improve functional recovery in animal models of SCI [51-53]. Activation of the Wnt/β-catenin pathway provides neuroprotective effects, preventing caspase-3 activation and mitochondrial dysfunction and suppressing neuronal apoptosis. Administration of Wnt agonists to experimental animals improved axonal regeneration. The anti-apoptotic and anti-inflammatory properties of the Wnt/β-catenin signaling pathway are exploited to protect neurons and glial cells [28,51].

In contrast, inhibiting the Wnt/β-catenin pathway may be beneficial in reducing inflammation and glial scar formation. Studies have shown that blocking β-catenin signaling can decrease the activation of pro-inflammatory microglia and reduce the formation of inhibitory glial scars [54]. Abrogation of β-catenin signaling in oligodendrocyte precursor cells reduces glial scarring and promotes axon regeneration after SCI [55]. By targeting the Wnt/β-catenin pathway, it may be possible to create a more favorable environment for axonal regeneration and functional recovery [56].

Pharmaceutical Administration

In SCI rat models, methylprednisolone has been shown to activate the Wnt/β-catenin signaling pathway, providing effective neuronal protection [57]. Notably, administration of methylprednisolone sodium succinate and methylprednisolone hemisuccinate in SCI promotes the expression of the anti-inflammatory gene PPARγ within the Wnt/β-catenin pathway, suppressing pro-inflammatory molecule levels, with the degree of inhibition appearing to be linked to the extent of Wnt pathway activation [58].

Simvastatin, a commonly used statin for lowering cholesterol, has shown potential neuroprotective effects in SCI management. In animal studies, it has been found to reduce neuronal apoptosis and improve the functional and pathological recovery via activating the Wnt/β-catenin signal pathway; however, the anti-apoptosis effects of simvastatin were reversed following suppressing the Wnt/β-catenin signaling pathway in primary spinal cord neurons [51]. Metformin is a widely used oral antidiabetic medication primarily prescribed for type 2 diabetes. Administration of metformin (50 mg/kg) enhanced motor functional recovery in rats after SCI, amplified β-catenin and brain-derived neurotrophic factor (BDNF) expression, suppressed neuron apoptosis and inflammatory response, and improved the recovery of pathological morphology at the SCI site by activating the Wnt/β-catenin signaling pathway [28].

Rapamycin, also known as sirolimus, is a macrolide compound with potent immunosuppressive, anti-proliferative, and neuroprotective properties [26]. Intraperitoneal injection of rapamycin in SCI rats increased the levels of β-catenin and BDNF in the injured spinal cord, decreased the loss of motor neurons, and enhanced motor functional recovery [52]. Melatonin is a hormone primarily produced by the pineal gland in the brain. It plays a crucial role in regulating the circadian rhythm and has additional antioxidant, immune-modulating, and neuroprotective properties. In rats, melatonin administration after SCI significantly upregulated the expression of the LRP6 receptor and β-catenin protein in the spinal cord, attenuated motor neuronal apoptosis, and improved the locomotor functions [59]. Following SCI, lithium, a mood stabilizer, has been found to inhibit GSK3β activity by inducing the expression of SGK1, leading to increased levels of β-catenin in the cytoplasm [53]. Nandrolone is a synthetic anabolic-androgenic steroid primarily used in the treatment of muscle wasting, osteoporosis, and anemia. Nandrolone reduces bone loss after SCI by modulating osteoclastic and osteoblastic activity and differentiation through the activation of Wnt signaling [60]. Polydeoxyribonucleotide, an adenosine receptor agonist that activates the Wnt pathway, systemically administered 1 hour following SCI, protected from tissue damage, demyelination, and reduced motor deficits by reducing the secretion of the pro-inflammatory cytokines TNF-α and IL-1β and attenuating apoptosis through the Wnt/β-catenin pathway [61].

Immunotherapy

Following SCI, early administration of BHQ880, a monoclonal antibody that targets DKK1, significantly improved motor functional recovery, induced preservation of myelinated tissue, and decreased astroglial and microglia/macrophage reactivity [11]. In SCI rats, intrathecal administration of a neutralizing antibody to Ryk resulted in considerable axonal growth of the corticospinal tract and promoted functional recovery [14].

Robust bone loss after acute motor-complete SCI can be prevented by sclerostin antibody, at least in part, by preserving osteocyte morphology and structure and related bone remodeling [62]. Sclerostin antibody does not prevent soleus muscle atrophy in rodents after SCI [63]. In SCI rodents, sclerostin inhibition via sclerostin antibody has been found to prevent SCI-induced cancellous and cortical bone deficits [64].

Cell Transplantation

According to the results of animal studies, cell transplantation therapies have a potential beneficial role for neuronal repair and functional plasticity. Transplantation of MSCs engineered to overexpress Wnt3a augments neural regeneration and functional recovery after SCI via the Wnt/Gli2 pathway [65]. Transplanted Wnt3a-secreting fibroblasts enhance axonal regeneration and functional improvement after SCI [66]. Wnt4-modified neural stem cell transplantation may repair the injured spinal cord and recover the motor dysfunction after SCI [67]. Transplantation of Wnt5a-modified BM-MSCs enhances recovery after SCI via the PI3K/AKT pathway [68]. TNF-α inhibition can block the ability of transplanted BM-MSCs in neurological functional recovery after SCI through the activation of the Wnt signaling pathway [69]. LINGO-1 shRNA promotes neural differentiation of neural stem cells and Wnt5a expression, probably by downregulating miR-15b-3p. Transplantation of LINGO-1 shRNA-treated neural stem cells promotes recovery of motor function after SCI [46].

Gene Therapy

By upregulating and silencing target genes, the Wnt protein family effectively attenuates inflammation, oxidative stress, and apoptosis, therefore triggering neuronal regeneration [38]. The increase of Wnt1 expression may enhance motor function recovery in SCI rats. However, Wnt1 protein alone cannot be highly expressed in the injured spinal cord over the long term. To address this, researchers used lentiviral vectors to sustain Wnt ligand expression. They found that prolonged Wnt1 overexpression through lentivirus-mediated Fz1 ligand delivery promotes myelin preservation and neuronal survival, decreases early astroglial reactivity and NG2+ cell gathering, and ameliorates motor function recovery in rats. These findings strongly support the therapeutic potential of sustaining high Wnt1 expression levels throughout SCI progression to treat motor dysfunction [21]. Long-term lentiviral-mediated Wnt1 overexpression in SCI rats improves motor functional recovery by increasing myelin preservation and neuronal survival and reducing early astroglial reactivity and NG2+ cell accumulation [20]. Another study by Zhao et al. demonstrated that the selective P2Y purinergic receptor agonist 2-MesADP can enhance motor recovery after acute SCI by promoting reactive astrocyte formation and stimulating oligodendrocyte proliferation by regulating gene expression via the Wnt/β-catenin pathway, which helps inhibit cellular apoptosis, promote myelin regeneration, and finally improve motor function recovery in SCI mice [70].

Use of Biomaterials

While therapeutic activation of Wnt/β-catenin shows potential, the risks of excessive activation include tumorigenesis and glial hyperreactivity. Studies highlight the importance of controlled delivery systems, such as nanoparticles, hydrogels, collagen scaffolds, and chitosan scaffolds, for local Wnt delivery, which mitigates systemic side effects [71]. The application of these biomaterials provides a stable environment for SCI repair and controls the effects of the Wnt/β-catenin signaling pathway, maximizing SCI treatment efficacy.

According to Su et al., decellularized extracellular matrix scaffolds can mimic the native 3D structure of the spinal cord, enhance the release of neurotrophic factors, and inhibit glial scar formation. Decellularized extracellular matrix scaffold seeded with adipose-derived stem cells enhances neuronal restoration and functional recovery after SCI through the regulation of the Wnt/β-catenin pathway [72]. Dental pulp stem cells/precursor cells may differentiate into neurons and glial cells. In combination with chitosan scaffolds, they accumulate total β-catenin levels, stimulating neuronal repair at the SCI site [73]. Alginate scaffolds promote the regeneration of axons, working with Wnt3a protein that augments the regeneration of the injured spinal cord [74]. A collagen microchannel scaffold carrying paclitaxel-liposomes elicits differentiation of neural stem cells into neurons through Wnt/β-catenin signaling for SCI repair [75].

Use of Exosomes

Exosomes are small extracellular vesicles, typically ranging from 30 to 150 nanometers in diameter, released by various cell types into the extracellular environment. They play a crucial role in intercellular communication by transferring bioactive molecules such as proteins, lipids, and nucleic acids, including microRNAs and mRNAs, between cells. Exosomes may promote neural repair and regeneration by delivering immunoregulatory factors, growth factors, neurotrophic factors, and angiogenic factors at the site of injury. Moreover, they can modulate the proliferation of neuron progenitor cells and their differentiation to neurons and oligodendrocytes, facilitating remyelination after SCI. Animal studies have found that, after SCI, exosomes derived from hUC-MSCs stimulate the LRP-6/Wnt/β-catenin pathway in neuronal cells, triggering the expression of c-myc and Cyclin D1 in spinal cord tissue, thereby exerting anti-apoptotic and anti-inflammatory effects [76]. In addition, exosomes from BM-MSCs can modulate the Wnt/β-catenin pathway in neuronal cells and suppress apoptosis [77]. Stem cell-originated extracellular vesicles can decrease the extent of SCI through the activation of the Wnt/β-catenin signaling pathway. In SCI mice, exosomes originated by M2 macrophages may stimulate the Wnt/β-catenin pathway in vascular endothelial cells at the injury site, increasing angiogenesis and neural function repair [44].

Use of Herbal Medicine

Resveratrol is a natural polyphenol found in certain plants, fruits, and red wine. In animal models, resveratrol could enhance the functional recovery and axonal regeneration, alleviate histological damage, and inhibit apoptosis following SCI via the regulation of the Wnt/β-catenin signaling pathway [78]. Treatment with resveratrol attenuates sublesional bone loss in SCI rats, suppresses inflammation, and restores Wnt/β-catenin signaling [79]. Protocatechuic aldehyde is a natural phenolic compound found in various medicinal plants, known for its antioxidant, anti-inflammatory, neuroprotective, and cardioprotective properties. It has been found to enhance the functional recovery of SCI by activating the Wnt/β-catenin signaling pathway [80]. Harpagide is a naturally occurring iridoid glycoside found in various medicinal plants that has been found to inhibit neuronal apoptosis and enhance axonal regeneration after SCI in rats through the activation of the Wnt/β-catenin signaling pathway [81].

Salvianolic acid B is a water-soluble polyphenolic compound found primarily in *Salvia miltiorrhiza* (Danshen), a traditional Chinese medicinal herb known for its potent antioxidant, anti-inflammatory, cardioprotective, neuroprotective, and anti-fibrotic properties. Following SCI, salvianolic acid B administration significantly reduced the expression of pro-apoptotic proteins in spinal cord tissues but promoted the expression of Bcl-2, an anti-apoptotic protein, through the activation of the Wnt/β-catenin signaling pathway [82]. Zhenbao Pill is a traditional Chinese medicine formula that suppresses neuronal damage caused by activated microglia after SCI through the miR-214-5p/SOX4/β-catenin axis [83]. Notoginsenoside R1 is a bioactive saponin found primarily in Panax notoginseng, a traditional Chinese medicinal herb, which alleviates SCI through the miR-301a/KLF7 axis to activate the Wnt/β-catenin pathway [84].

Use of Electrical Stimulation

Electrical stimulation has been shown to promote the activation of the Wnt/β-catenin pathway in neurons, glial cells, and stem cells. This activation can enhance neurogenesis, axonal growth, and synaptic plasticity, which are crucial for spinal cord repair. Electrical stimulation can enhance neural stem cell proliferation and differentiation, partly through Wnt/β-catenin signaling. Combining electrical stimulation with Wnt/β-catenin modulation offers a promising therapeutic strategy for SCI. Preclinical studies suggest that electrical stimulation enhances functional recovery, possibly by synchronizing Wnt signaling activation with neuroplasticity mechanisms [85-88].

Electrical stimulation has been found to promote functional recovery after SCI by activating endogenous spinal cord-derived neural stem/progenitor cells while increasing Wnt3, Wnt7, and β-catenin protein levels [86]. Moreover, electric field stimulation triggers differentiation of neural stem cells into neurons via PI3K/Akt/GSK-3β/β-catenin activation for SCI treatment [87]. In SCI rats, via Wnt/β-catenin signaling, electrical stimulation regulates genes for the motor endplate and calcium binding in muscle [88].

In SCI models, pulsed electromagnetic fields enhanced BDNF and vascular endothelial growth factor (VEGF) expression in BM-MSCs through the Wnt/β-catenin signaling pathway [89]. According to Shao et al., pulsed electromagnetic fields mitigate skeletal deterioration in bone mass, microarchitecture, and strength by promoting canonical Wnt/β-catenin signaling-mediated bone formation in SCI rats [90].

Use of Acupuncture

Electroacupuncture at GV14 and GV4 exhibits neuroprotective effects against SCI by upregulating Wnt1, Wnt3a, and β-catenin expression [91]. Similarly, electroacupuncture at the Dazhui and Mingmen acupuncture points promoted the proliferation and differentiation of neural stem cells after SCI by significantly increasing the expression levels of Wnt1 and β-catenin [92]. Fire needle acupuncture promoted the neuronal differentiation of neural stem cells after SCI by regulating Wnt/ERK multiple pathways [93].

Discussion

The evidence compiled in this review underscores the central and multifaceted role of the Wnt/β-catenin signaling pathway in SCI pathology and recovery. Yet, beyond describing its involvement in inflammation, apoptosis, neuroprotection, and regeneration, deeper questions remain about its precise temporal and spatial modulation, its crosstalk with other pathways, and the contextual nuances that dictate whether its activation is beneficial or detrimental. One of the most compelling findings from the reviewed literature is the dual nature of the Wnt/β-catenin pathway. Canonical Wnt signaling is neuroprotective and pro-regenerative in early and subacute stages of SCI, yet its prolonged activation can contribute to maladaptive processes such as glial scarring or aberrant synaptogenesis, which may intensify chronic pain or impede axonal regrowth. This duality indicates that successful therapeutic strategies must finely tune Wnt signaling in a spatiotemporal and cell-specific manner.

Wnt/β-catenin signaling does not operate in isolation. It intersects with several critical pathways, including PI3K/Akt, mTOR, Notch, and TGF-β. A systems biology approach is necessary to decipher how Wnt signaling hierarchically interacts with these networks during injury progression and repair. For instance, modulating Wnt may inadvertently alter angiogenic or inflammatory processes via indirect influence on VEGF or NF-κB, suggesting an underappreciated complexity in designing combinatorial or multi-targeted therapies.

Emerging evidence suggests that distinct cellular responses to Wnt activation (e.g., astrocytes vs. oligodendrocyte precursor cells vs. microglia) may underpin the conflicting outcomes observed in experimental models. Tailoring Wnt pathway modulation to specific cell types using targeted gene delivery systems (e.g., viral vectors and nanoparticle-mediated RNA interference) may increase therapeutic specificity and minimize off-target effects. This approach could facilitate controlled remyelination, axonal sprouting, and immune suppression without triggering undesirable astroglial hyperplasia or chronic pain sensitization.

Nearly all experimental evidence to date arises from rodent models, which differ markedly from human spinal cord structure, injury mechanisms, and immune responses. Moreover, many interventions (e.g., lentiviral overexpression and biomaterial scaffolds) pose safety and scalability concerns for human application. Future preclinical models, including large animals and human organoids, could offer more predictive insights. Additionally, integrating Wnt-focused biomarker profiling into early-phase clinical trials may help identify responders and optimize dosing windows.

The role of microRNAs in modulating Wnt signaling post-SCI is a promising but still underexploited frontier. Several miRNAs have shown potential in orchestrating Wnt activity to protect against neuronal apoptosis and enhance stem cell function. Coupled with exosome-based delivery systems, this endogenous regulatory machinery could be harnessed to achieve highly targeted and minimally invasive interventions. Notably, exosome-mediated Wnt activation may circumvent the oncogenic risks of systemic Wnt ligand administration.

## Conclusions

The Wnt/β-catenin signaling pathway plays a multifaceted role in the pathogenesis of SCI and offers a promising avenue for therapeutic intervention. The canonical Wnt/β-catenin signaling pathway primarily enhances functional recovery and injury repair of neural stem cells by regulating cell proliferation, differentiation, and apoptosis. Moreover, it promotes angiogenesis, tissue repair, and axonal regeneration. In contrast, the non-canonical Wnt/β-catenin signaling pathway modulates cell polarity and migration. The subsequent role of the Wnt/β-catenin signaling pathway in SCI pathogenesis brings new hope for neural repair following SCI, as many treatment modalities, such as pharmaceutical administration, stem cell transplantation, electric stimulation, the use of biomaterials, and exosomes, exert beneficial effects in SCI animal models by interacting with the Wnt/β-catenin signaling pathway. Although significant progress has been made, further studies are needed to improve our understanding of its context-dependent effects and to address therapeutic challenges.
